# Altered regional brain function in the treatment‐naive patients with somatic symptom disorder: a resting‐state fMRI study

**DOI:** 10.1002/brb3.521

**Published:** 2016-07-24

**Authors:** Qiang Li, Yong Xiao, Yinghui Li, Lei Li, Na Lu, Zhi Xu, Xiaodong Mou, Shenqin Mao, Wei Wang, Yonggui Yuan

**Affiliations:** ^1^ Department of RadiologyTangdu Hospitalthe Fourth Military Medical UniversityXi'anChina; ^2^ Department of NeurosurgeryFuzhou General HospitalXiamen UniversityFuzhouChina; ^3^ Department of Psychosomatics and PsychiatryAffiliated Zhongda HospitalMedical School of Southeast UniversityNanjingChina

**Keywords:** functional magnetic resonance imaging, medial prefrontal cortex, regional homogeneity, resting state, somatic symptom disorder

## Abstract

**Introduction:**

Somatic symptom disorder (SSD) is an illness that occurs over a long time and results in significant disruption in daily life. Clinically, SSD patients typically express complaints that involve a variety of organ systems. However, the neural mechanism of SSD remains poorly understood.

**Methods:**

Using resting‐state functional magnetic resonance imaging, we investigated the characteristics of the regional basal brain function during resting state in patients with SSD. Eleven treatment‐naïve SSD patients and 12 age‐matched healthy controls were recruited in this study. Between‐group differences in regional homogeneity values were analyzed.

**Results:**

Compared with the healthy control group, the SSD group showed significant increases in regional homogeneity values in the right medial prefrontal cortex, anterior cingulate cortex and supramarginal gyrus, and significant decreases in the bilateral middle occipital gyrus, superior occipital gyrus and right cuneus and left postcentral gyrus and cerebellum. Meanwhile, the regional homogeneity value of the right medial prefrontal cortex positively correlated with the total duration of SSD.

**Conclusions:**

The abnormal resting‐state patterns in regional brain activity may contribute to understanding the mechanism of SSD.

## 1 Introduction

Somatic symptom disorder (SSD) is a new category in DSM‐5 (Dimsdale & Levenson, 2013). It is one of the most common mental disorders in general hospital and primary care. The prevalence of SSD in the general adult population is about 5%–7% (American Psychiatric Association 2013). SSD patients typically have multiple somatic symptoms that are distressing, resulting in significant disruption of daily life, although sometimes only one severe symptom, most commonly pain, is present. Up to date, the diagnosis of SSD is largely based on clinical signs and symptoms. The neural mechanism of SSD remains poorly understood.

Despite increasing evidence for roles of structural and functional brain alterations in the neural mechanism of mental disorders, there were only a few neuroimaging studies that focused on SSD. Atmaca and his colleagues (Atmaca, Sirlier, Yildirim, & Kayali, 2011) found that, compared to healthy controls, the patients with somatization disorder showed significantly smaller mean volumes of the bilateral amygdala. Hakala et al. (Hakala et al., 2004) found that patients with somatization disorder showed bilateral enlargement of caudate nuclei volumes when compared with the healthy controls. Using functional magnetic resonance imaging (fMRI) with an emotional empathy task, de Greck et al. (de Greck et al., 2012) found that patients with somatoform disorder demonstrated lower activity in several regions including bilateral parahippocampal gyrus, left amygdala, left postcentral gyrus, left superior temporal gyrus, left posterior insula, and bilateral cerebellum. The neuroimaging plays an important role in providing evidence about the possible mechanisms underlying SSD (Browning, Fletcher, & Sharpe, 2011; Coghill, McHaffie, & Yen, 2003; de Greck et al., 2011, 2013).

Since SSD is a chronic disorder that distress patients for years, and the deficit in task‐related brain activity may be based on the deficit in basal brain function, we hypothesize such distress would cause alterations in basal brain functions. Resting‐state functional connectivity is new neuroimaging technique that assesses spontaneous ongoing brain activity without engaging subject into any explicit tasks. Such spontaneous activity exhibits distinct spatial and temporal patterns, forming the so‐called functional networks. Converging data demonstrate that alterations in brain networks are implicated in a number of neuropsychiatric disorders, including depression, schizophrenia (Hasler & Northoff, 2011). Given that literature data suggesting multiple brain regions and systems implicated in SSD, in this study, we applied regional homogeneity (REHO), a resting‐state fMRI data analysis approach to assess synchrony of spontaneous activity in neighboring imaging voxels (Zang, Jiang, Lu, He, & Tian, 2004), and conducted whole‐brain search. This method does not require a prior knowledge in brain systems involved in SSD, and appears particularly suitable for identifying dysfunctions in brain systems implicated in SSD.

## 2 Method

### 2.1 Participants

A total of 11 patients (four males and seven females) were recruited from the Department of Psychosomatics and Psychiatry of ZhongDa Hospital, Southeast University, China. All patients were treatment‐naïve, and met the diagnostic criteria for SSD (DSM‐5). The diagnosis was confirmed by two professional psychiatrists. Besides meeting SSD criteria, all patients met the following inclusion criteria: (1) over 18 and below 60‐years old; (2) right‐handed; (3) Han Chinese ethnics and exclusion criteria: (1) with other major psychiatric illness, including depression, anxiety, substance abuse, or dependence; (2) with primary neurological illness, including dementia or stroke; and (3) with any white matter changes such as infarction or other vascular lesions detected by T2‐weighted MRI. Among the SSD patients, seven patients complained headache, five patients complained dizziness, four patients complained back pain, and four patients complained abdominal pain. One patient had a diagnosis of gastritis, one patient had Sjogren's syndrome, and two patients had hypertension which was diagnosed by related physicians. The depression and anxiety symptoms of the SSD patients were measured by 17‐item Hamilton Depression Scale (HAMD) (Hamilton, 1960) and Hamilton Anxiety Scale (HAMA) (Hamilton, 1959). Twelve healthy controls were also recruited from the community (six males and six females). Healthy controls had no history of psychiatric disorder. They also met the inclusion and exclusion criteria. The demographical data of all subjects are shown in Table 1. No significant differences in age, gender, or education were observed between SSD patients and healthy controls except for HAMA and HAMD score. All subjects gave their written informed consent after the procedure had been carefully explained. The research was approved by the Research Ethics Committee of Affiliated ZhongDa Hospital, Southeast University.

**Table 1:** Demographic and clinical characteristics of SSD patients and healthy control subjects

	SSD (*n* = 11)	HC (*n* = 12)	χ^2^/*t* value	*p* value
Gender (male/female)	4/7	6/6	0.434	.510
Age (years)	49.5 ± 12.1	49.0 ± 14.6	0.097	.924
Education (years)	10.6 ± 4.8	12.8 ± 2.9	−1.344	.193
Age of onset (years)	45.9 ± 11.6	–	–	–
Course (months)	43.9 ± 46.4	–	–	–
Family history (%)	0 (–)	0 (–)	–	–
HAMA	16.2 ± 5.9	1.1 ± 1.0	13.894	<.001
HAMD	18.8 ± 4.1	1.8 ± 1.1	8.338	<.001

SSD, Somatic symptom disorder; HC, Healthy control group; HAMA, Hamilton Anxiety Scale; HAMD, Hamilton Depression Scale.

### 2.2 fMRI data acquisition

Scanning took place on the 3.0T Siemens verio scanner at Zhongda Hospital. During the scanning, each subject was instructed to keep still, rest with their eyes closed but not fall asleep and not to think about anything specifically. Ear plugs and foam padding were used to reduce noise and minimize head movement. The functional images were collected using a gradient Echo Planar Imaging (GRE‐EPI) sequence (repetition time = 2,000 ms, echo time = 25 ms, number of slices = 36, slice thickness = 4 mm, gap = 0 mm, imaging matrix = 64 × 64, flip angle = 90°, field of view = 240 × 240 mm^2^) with in‐plane resolution of 3.75 × 3.75 mm^2^. For each subject, 240 echo‐planar volumes were collected, respectively, during the resting‐state run. The total acquisition duration lasted for 8 min. The corresponding high‐resolution 3D T1‐weighted images were also collected for spatial normalization purpose. The structural images were reviewed by an experienced radiologist to ensure the absence of structural abnormalities in these subjects.

### 2.3 Data processing

The fMRI data processing was conducted using Statistical Parametric Mapping (SPM) 8 software (http://www.fil.ion.ucl.ac.uk/spm). For each subject, the first five volumes were discarded to avoid transient signal changes before magnetization reached steady state. The remaining volumes were slice time corrected with reference to the middle slice, corrected for motion by realignment to the first volume, registered to the 3D T1‐weighted images, normalized to a standard SPM T1 template, then spatially normalized to a standard SPM EPI template. The images were resampled to 3 × 3×3 mm^3^. The subjects with head motion more than 2 mm maximum displacement in any direction of *x*,* y,* and *z* or 2° of any angular motion throughout the course of scan were excluded. Furthermore, data preprocessing and REHO analysis were performed with REST software (http://resting-fmri.sourceforge.net). Subsequent data preprocessing included removal of linear trends and temporally filtered (band pass, 0.01–0.08 Hz) to remove the effects of very low‐frequency drift and high‐frequency noises (e.g., respiratory and cardiac rhythms) (Biswal, Yetkin, Haughton, & Hyde, 1995; Lowe, Mock, & Sorenson, 1998). The REHO calculation procedure was described in the previous study (Zang et al., 2004). Briefly, this was accomplished on a voxel‐by‐voxel basis by calculating the Kendall's concordance coefficient of the time series of a given voxel and that of its nearest 26 neighbors. A larger value for a given voxel represents a higher regional homogeneity within a cluster made up of the voxel and its nearest neighbors. Therefore, we derived an individual REHO map in voxel‐wise manner. Finally, the resulting data were smoothed with an isotropic 6‐mm full‐width half‐maximum Gaussian kernel.

### 2.4 Statistical analysis

A two‐sample *t*‐test was performed to determine REHO differences in brain regions between SSD and healthy control groups. The head motions, age, gender, and education were taken into account as covariates. For all of the whole‐brain contrasts, a voxel level significance threshold corresponding to *p *<* *.005 was set in combination with cluster level correction for false positives (*p *<* *.05) based on 10,000 Monte Carlo simulations (Cox, 1996). All coordinates reported were in the Montreal Neurological Institute space. To explore the relationship between the REHO characteristics and duration of SSD, we extracted REHO values from brain regions showing significant difference between the two groups and performed Pearson correlation analyses with duration of SSD. The age and years of education were taken as covariates. Significance was set at *p *<* *.05.

## 3 Results

### 3.1 Differences in REHO value between SSD patients and healthy controls

**Figure 1:**
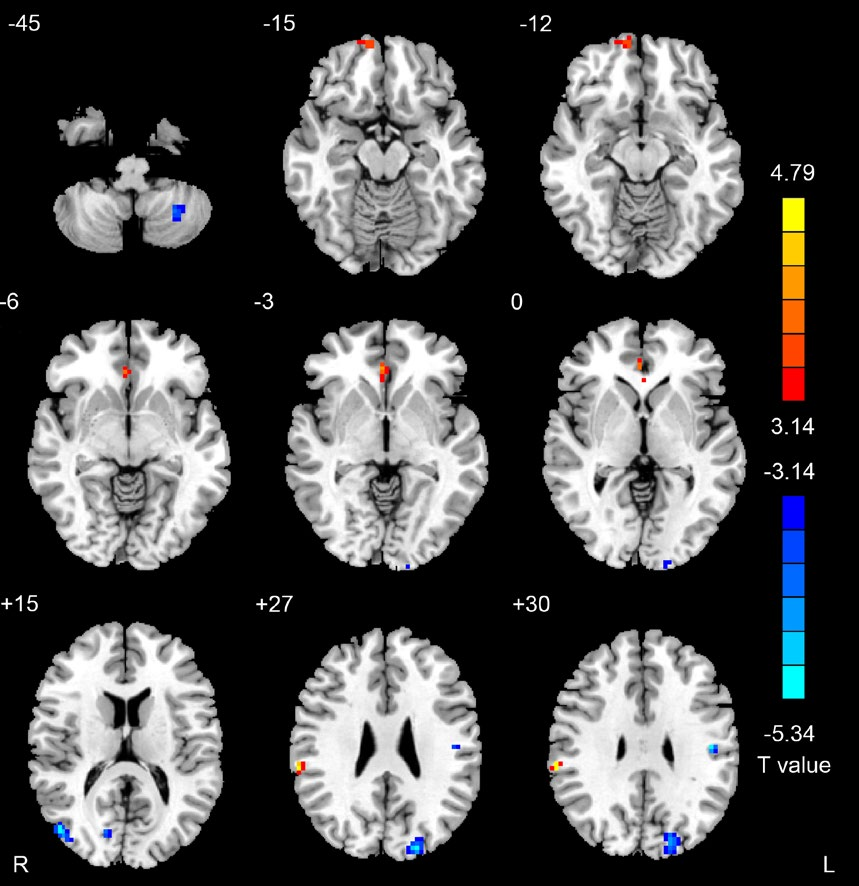
The differential regions of REHO value between SSD and HC group (*p *<* *.05, corrected for Monte Carlo simulations; ACC, anterior cingulate cortex; MPFC, medial prefrontal cortex; R, right; L, left)

Compared to the healthy control group, the SSD group showed significant increases in REHO values in the right medial prefrontal cortex (MPFC), anterior cingulate cortex (ACC), and supramarginal gyrus; significant decreases in REHO values were seen in the bilateral middle occipital gyrus, superior occipital gyrus and right cuneus and left postcentral gyrus and cerebellum (Fig. 1 and Table 2).

**Table 2:** REHO difference between SSD and healthy control group (*p *<* *.05, corrected for Monte Carlo simulations)

Brain regions		Brodmann's area	Peak location			Peak *t*‐score	Number of voxels
			x	y	z		
MPFC	R	11	9	60	−18	3.78	18
ACC	R	10, 11	3	36	−3	4.17	14
Supramarginal gyrus	R	2	63	−33	27	4.78	13
	R	19	45	−81	12	−5.33	45
Middle occipital gyrus	L	19	−42	−84	6	−4.85	15
		17	−18	−99	3	−3.74	12
Superior occipital gyrus	R	19	27	−81	39	−4.52	12
	L	19	−21	−90	27	−5.59	47
Cuneus	R	17	15	−78	12	−4.23	12
Postcentral gyrus	L	2	−30	−45	60	−4.78	12
		48	−48	−218	30	−5.16	12
Cerebellum	L	–	−30	−57	−45	−4.22	23

MPFC, Medial prefrontal cortex; ACC, Anterior cingulate cortex; R, right; L, left.

### 3.2 Correlations

**Figure 2:**
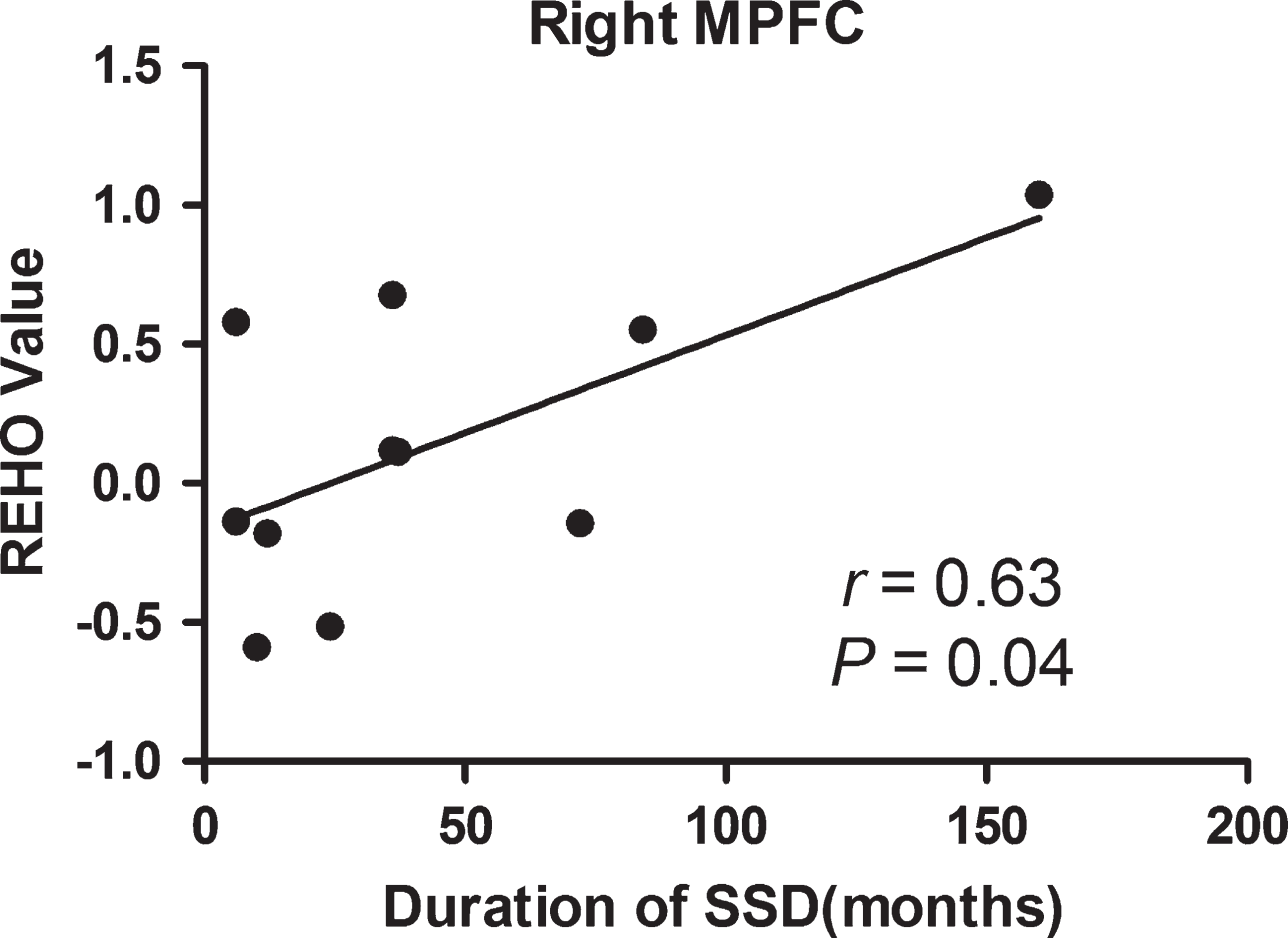
Correlation map between the REHO value of right MPFC and duration of SSD in the patient group (*r *=* *0.63, *p *=* *.04, MPFC, medial prefrontal cortex)

We analyzed the relationship between the durations of SSD and the REHO values in the above‐mentioned brain regions that showed significant difference between the two groups. Among them, only the right MPFC showed a significant positive correlation (*r *=* *.63, *p *=* *.04; Fig. 2). No significant correlations were found in other brain regions.

## 4 Discussion

In this study, we found abnormal changes in regional basal brain activity in treatment‐naive SSD patients. Areas showing significant increases in REHO values were distributed mainly in the MPFC, ACC, and supramarginal gyrus; areas showing decreased REHO values were in the middle occipital gyrus, superior occipital gyrus, cuneus, postcentral gyrus, and cerebellum.

The MPFC is a key brain structure in fronto‐limbic circuit networks. It integrates both emotional and cognitive information, and functions as a mediator of fronto‐limbic circuit regulation (Mayberg et al., 1999; Seminowicz et al., 2004). The ACC plays an important role in the evaluation, processing, and integration of sensory, motor, cognitive, and emotional aspects (Caetano et al., 2006; Rainville, Duncan, Price, Carrier, & Bushnell, 1997). According to the view of a top‐down inhibitory (Petrovic & Ingvar, 2002; Valet et al., 2004), we speculated that the abnormally increased REHO value of the MPFC and ACC in SSD patients may point to the dysfunction of emotion regulation. The positive correlation between REHO value of the MPFC and total duration of SSD further suggests that the accumulated damage in MPFC and the accumulated abnormality in the emotional processing. For example, a meta‐analysis of longitudinal MRI studies found that progressive cortical gray matter alterations in schizophrenia occur with regional and temporal specificity (Vita, De Peri, Deste, & Sacchetti, 2012). Cumulative brain abnormalities during a long term might be involved in neural alteration of psychiatric disorders (Mocking et al., 2016; Silk et al., 2016). The supramarginal gyrus is essential for visuospatial processing (Lemche et al., 2013). Recent findings have indicated that the supramarginal gyrus is active in emotion attribution in normal controls (Sommer et al., 2010). The increased REHO value in SSD patients might suggest that these patients are involved in abnormally increased emotion attribution.

We also observed decreased REHO value in the left postcentral gyrus and cerebellum, right cuneus and bilateral occipital gyrus. The postcentral gyrus is known as the first somatosensory cortex of primates, which also plays a role in the visual recognition and induction of emotion (Adolphs, Damasio, Tranel, Cooper, & Damasio, 2000; Rudrauf et al., 2009). The decrease in REHO value in the postcentral gyrus may indicate the deficiency of providing the somatosensory information necessary for emotion generation and emotion experience. The occipital gyrus and cuneus are thought to relate with visual recognition circuit (Tao et al., 2013). A SPECT study demonstrated that patients with somatoform disorder showed slower rCBF in bilateral occipital lobes relative to the control group (Karibe et al., 2010). These structures might be engaged in some aspects of uncomfortable experience in SSD patients. Traditionally, the cerebellum is considered to coordinate motor behavior (Stein & Glickstein, 1992). However, more recent evidence suggests that the cerebellum is involved in emotional processing (Schmahmann, 2010; Schutter & van Honk, 2005). It also plays a role in the processing of autobiographic memory (Svoboda, McKinnon, & Levine, 2006). The decreased REHO value might indicate disturbed procession of emotion memory to some extent in SSD patients.

Recently, another resting‐state fMRI study in patient with somatoform pain disorder suggested that these patients exhibited atypical precentral gyrus activation compared with the healthy controls (Yoshino et al., 2014). However, the result is inconsistent with what we found. The reason for the discrepancy is likely to be heterogeneity of the populations. Among our SSD patients, most of them complain of uncomfortable experience but not pain‐related symptoms. Therefore, future study on the differences in regional neural activity between subtypes of SSD patients is warranted.

Altogether, we speculated that the abnormal REHO value changes in the above brain regions might be related to the constantly increased uncomfortable experience and point to a defective somatosensory regulation in patients suffering from SSD. However, further studies are needed to get a better understanding of the mechanism these abnormal activity patterns.

A number of caveats apply to this study. First, the sample was relatively small. A larger sample size would be necessary to confirm the current results in future studies. Second, although all of the participants denied falling asleep during MRI scan, the inability to control subjects’ thoughts during imaging was a limitation common in resting‐state fMRI studies. Third, considering very few reliable replicated findings have been revealed by previous fMRI studies with various somatization syndromes, different primary complaints (pain or dizziness) in our study may limit our understanding of common neural function alterations in SSD. Fourth, as SSD and some concurrent medical illness frequently occur together (American Psychiatric Association 2013), we could not rule out the effect of general medical condition.

Our study demonstrated the abnormal resting‐state patterns in regional brain activity in SSD patients. The results suggested that patients with SSD have abnormal baseline function of brain activity. In addition, the REHO method may be potentially helpful in understanding the mechanism of SSD.

## Funding Information

This work was supported by the grants from the National Natural Science Foundation of China (Nos. 30970814, 81371488, and 81201081).

## Conflict of Interest

None declared.
